# Bevacizumab in recurrent WHO grades II–III glioma

**DOI:** 10.3389/fonc.2023.1212714

**Published:** 2023-07-18

**Authors:** Soufyan Annakib, Valérie Rigau, Amélie Darlix, Catherine Gozé, Hugues Duffau, Luc Bauchet, Marta Jarlier, Michel Fabbro

**Affiliations:** ^1^ Department of Medical Oncology, Institut Régional du Cancer de Montpellier, University of Montpellier, Montpellier, France; ^2^ Department of Medical Oncology, CHU de Nîmes, University of Montpellier, Nimes, France; ^3^ Department of Pathology and Onco-biology, CHU de Montpellier, University of Montpellier, Montpellier, France; ^4^ Institut de Génomique Fonctionnelle, INSERM, CNRS, University of Montpellier, Montpellier, France; ^5^ Faculty of Medicine, University of Montpellier, Montpellier, France; ^6^ Department of Neurosurgery, CHU de Montpellier, University of Montpellier, Montpellier, France; ^7^ Department of Biostatistics, Institut Régional du Cancer de Montpellier, University of Montpellier, Montpellier, France

**Keywords:** anaplastic glioma, astrocytoma, oligodendroglioma, recurrent glioma, transformed low grade glioma, bevacizumab

## Abstract

**Purpose:**

The management of recurrent WHO grades II–III (rGII–III) glioma is not well established. This study describes the clinical outcomes in patients who received bevacizumab as rescue treatment.

**Methods:**

In this retrospective study, the main inclusion criteria were as follows: adult patients with histologicaly proved rGII–III glioma according 2016 WHO classification treated with bevacizumab from 2011 to 2019, T1 contrast enhancement on MRI. Efficacy was assessed using the high-grade glioma 2017 Response Assessment in Neuro-Oncology criteria. Progression-free survival (PFS) and overall survival (OS) were estimated using the Kaplan–Meier method.

**Results:**

Eighty-one patients were included (M/F ratio: 1.7, median age at diagnosis: 38 years) among whom 46 (56.8%) had an initial diagnosis of grade II glioma. Previous treatments included at least one surgical intervention, radiotherapy (98.8%), and ≥ 2 chemotherapy lines (64.2%). After bevacizumab initiation, partial response, stable disease, and progressive disease were observed in 27.2%, 22.2%, and 50.6% of patients. The median PFS and OS were 4.9 months (95% confidence interval [CI] 3.7–6.1) and 7.6 months (95% CI 5.5–9.9). Bevacizumab severe toxicity occurred in 12.3%. Twenty-four (29.6%) patients discontinued bevacizumab without radiological progression. Oligodendroglioma and age ≥ 38 years at diagnosis were more frequent in this subgroup (odds ratio = 0.24, 95% CI 0.07–0.84, *p* = 0.023 and 0.36, 95% CI 0.13–0.99, *p* = 0.042). Ten of these 24 patients were alive at 12 months and two patients at 8 years after bevacizumab initiation, without any subsequent treatment.

**Conclusion:**

Bevacizumab can be an option for heavily pretreated patients with rGII–III glioma with contrast enhancement. In our study, bevacizumab displayed prolonged activity in a subgroup of patients.

## 1 Introduction

In the United States, diffuse low-grade or anaplastic gliomas represent 22.6% of all diagnosed gliomas (1, 2). In France, the standardized incidence rate adjusted on the United States and world population for all grades II–III glioma is 16.0/1 million individuals (3). Since the 2016 WHO classification, molecular features have been included to better classify diffuse gliomas (4). The entity of oligoastrocytoma was deleted compared with the 2007 WHO classification. New entities are diffuse astrocytoma isocitrate dehydrogenase mutated (IDHm) or wild type, oligodendroglioma IDHm, and 1p19q codeleted. Anaplastic gliomas are classified as grade III and non-anaplastic as grade II (4). However, since 2022, the new WHO classification has added more genetic mutations that classify diffuse astrocytoma as grade IV gliomas (homozygous *CDKN2A/B* deletion, *TERT* promoter mutation, *EGFR* gene amplification, and chromosome 7 gain/chromosome 10 loss). These mutations were not systematically used for glioma classification in the WHO 2016 classification (2). Low-grade glioma is characterized by a natural progression toward more aggressive disease (grades III and IV) often characterized by the occurrence of contrast enhancement on MRI (5). In patients with grades II–III glioma, overall survival (OS) is heterogenous reaching almost 20 years in some cases (6–8).

Grades II–III glioma management is not standardized (9). Adjuvant treatment with chemotherapy (temozolomide) or combined chemotherapy (e.g., Procarbazine-CCNU-Vincristine [PCV]) and/or radiotherapy can be proposed upfront to selected patients (10). Similarly, the management of recurrent grades II–III glioma is not standardized. Chemotherapy can be proposed at disease relapse, but only limited data support this strategy (10). Moreover, new treatment modalities are needed due to chemotherapy-induced resistance (11).

The use of anti-angiogenic agents in glioma is based on neovascularization driven mainly by vascular endothelial growth factor (VEGF) signaling and its endothelial receptor VEGFR2 (12). Bevacizumab, a monoclonal antibody against VEGF, has demonstrated clinical benefit in glioblastoma in phase II trials, but not in phase III trials (13–17). Bevacizumab associated with temozolomide showed poor efficacy compared with temozolomide alone in patients with first recurrence of grades II–III glioma without 1p19q codeletion (18). Retrospective studies suggest that bevacizumab-based combination treatments or bevacizumab alone may be a strategy for alkylating agent-refractory grade III glioma, defined as progressive disease after temozolomide (19–22). Few prospective studies (phase II) showed a potential interest of bevacizumab as rescue treatment for recurrent grade III glioma (23, 24). Altogether, these results suggest that bevacizumab may be proposed as rescue treatment in recurrent grades II–III glioma, specifically to heavily pretreated patients (22). However, data on bevacizumab use in recurrent grade II glioma are scarce. Moreover, no established biomarker of the response to bevacizumab is available. Clinical features (performance status and hypertension), tumor profile (proneural subtype, VEGF, VEGFR, and carbonic anhydrase IX expression), tumor mutational status (*TP53*, *IDH1*, *MGMT*, and 1p19q codeletion), and circulating biomarkers (matrix metalloproteinases 2 and 9) have been reported as possible bevacizumab efficacy biomarkers in malignant glioma (25–33).

However, these studies presented limitations and results were conflicting. In routine practice, some patients with recurrent grades II–III glioma who received bevacizumab seem to display long-term benefits, even after its discontinuation. Indeed, a retrospective study of 435 patients with recurrent grades II and III glioma identified 22 patients (eight grade II and 14 grade III) who were alive 3 years after initiation of bevacizumab. However, in this study, recurrence was not clearly defined. Prior treatment history or radiologic criteria were not considered before bevacizumab treatment (e.g., bevacizumab was administered at first recurrence in 10 patients) (34).

Here, we were interested in bevacizumab efficacy in heavily pretreated grades II–III recurrent glioma patients. T1 contrast enhancement was used as a marker for disease malignant transformation. The aim of this retrospective study was, therefore, to describe clinical outcomes after starting treatment with bevacizumab and to determine the patient and tumor characteristics associated with the efficacy of bevacizumab across the whole population. The secondary endpoint was to describe patients who had discontinued bevacizumab without radiographic progression and to examine associated patient characteristics.

## 2 Materials and methods

### 2.1 Study design

This retrospective study included patients treated with bevacizumab for recurrent grades II–III glioma and contrast enhancement on MRI at our institution from January 2011 to December 2019. The study was conducted in accordance with the World Medical Association Declaration of Helsinki. The retrospective data collection was conducted in accordance with the MR004 Reference Methodology established by French national “information and liberties” council (CNIL). While no approval from the national Ethical Committee is required, the data analysis has been internally documented and referenced as UB-2019-042. Due to the study retrospective nature, patient consent was not deemed necessary, in accordance with French regulations.

### 2.2 Clinical and biological features

Recurrent glioma was defined as any recurrence after local intervention (one surgical intervention and one radiotherapy) and one systemic chemotherapy treatment. Increased gadolinium signal on MRI T1 images was mandatory for inclusion. Clinical data were collected from health records (by S.A.). Tumors were classified according to the WHO 2016 classification. 1p19q codeletion status was obtained using either fluorescent *in situ* hybridization or comparative genomic hybridization array. The IDH mutation (IDHm) status and α thalassemia/mental retardation X-linked (ATRX) protein expression (if the 1p19q codeletion status was unknown) were reviewed by immunohistochemistry (IHC), whenever possible, using the first tissue sample (by V.R.). Indeed, it has been established that 1p19q codeletion and *ATRX* gene loss are mutually exclusive, and IHC to detect ATRX protein expression is a good surrogate for genetic analysis of *ATRX* gene mutations (35–38). Hereafter, “1p19q codeletion” will be used to define tumors with 1p19q codeletion or positive ATRX protein expression.

### 2.3 Study outcomes

Efficacy was assessed according the 2017 Response Assessment in Neuro-Oncology (RANO) criteria for high grade gliomas (due to T1 contrast enhancement) using clinical and brain MRI data (digital images if available or MRI report) every 3 months (39). Bevacizumab tolerance was assessed according to the Common Terminology Criteria for Adverse Events (CTCAE) 5.0 classification.

OS was defined as the time from starting bevacizumab to death from any cause or the last follow-up date. Progression-free survival (PFS) was defined as the time from bevacizumab initiation to disease progression, death from any cause, or last follow-up date.

### 2.4 Statistical analysis

Demographic, baseline disease characteristics and treatments were described. The Kaplan–Meier method was used to analyze survival data (OS and PFS). Survival distributions were compared with the log rank test. Hazard ratios (HRs) and their 95% confidence intervals (CIs) were estimated using a Cox proportional hazard model.

Predictive factors of bevacizumab response were investigated using a logistic regression model. Clinically relevant variables and variables with *p*-value < 0.3 in the univariate analysis were included in the multivariate analysis. Odds ratios (ORs) were presented with their 95% CI.

No imputation method was used for missing data. All statistical tests were two-sided and the significance level was set at 5%. Statistical analyses were performed with STATA v16.0 software.

## 3 Results

### 3.1 Baseline patient characteristics

Eighty-one patients (63% men and 37% women) with recurrent grades II–III glioma received bevacizumab between 2011 and 2019. Their baseline characteristics are summarized in **Table 1**. At diagnosis, 46 (56.8%) patients had grade II and 35 (43.2%) grade III glioma, and their median age was 38 years [range 17.5–72.7]. IDHm was detected in 43 (53.2%) patients and 1p19q codeletion in 15 (18.5%). According to the WHO 2016 classification, the main histological forms were astrocytoma IDHm in 27 (33.3%) patients and oligodendroglioma IDHm/1p19q codeletion in 13 (16%) patients. Excluding missing data, the proportion of IDHm was 63%. The tumor mutation status of 26 patients could not be confirmed due to insufficient tissue material. Among them, 13 had oligodendroglioma according to the WHO 2007 classification.

**Table 1:** Patients’ baseline characteristics.

Variable		Whole sample (*n* = 81)	Patients alive at 1 year after BEV discontinuation without radiological progression ^(a)^ (*n* = 10)
**Bevacizumab**		81 (100)	10 (100)
**Median age at diagnosis, years (range)**		38 (17.5 to 72.7)	39.9 (19.6 to 48.4)
**Sex (male/female)**		51/30 (63/37)	5/5 (50/50)
WHO Performance Status at BEV initiation			
0–1		42 (51.9)	7 (70)
2		25 (30.9)	2 (20)
3–4		14 (17.3)	1 (10)
Initial tumor location			
Frontal		31 (38.3)	4 (40)
Temporal		28 (34.6)	2 (20)
Other		22 (27.1)	4 (40)
Initial tumor histology (WHO 2016 classification)			
Astrocytoma (grades II–III) IDHm		27 (33.3)	2 (20)
Astrocytoma (grades II–III) IDHwt		15 (18.5)	1 (10)
Oligodendroglioma (grades II–III) IDHm 1p19q codeletion		13 (16)	4 (40)
Not reclassified ^(b)^		26 (32.1)	3 (30)
WHO tumor grade at latest surgery			
Upgrading from grade II to grade III		7 (8.6)	3 (30)
Upgrading from grade III to grade IV		5 (6.2)	0 (0)
Upgrading from grade II to grade IV		4 (4.9)	1 (10)
Unchanged		8 (9.9)	0 (0)
Unknown/No additional surgery		57 (70.4)	6 (60)
Histological WHO 2016 grade at diagnosis			
II		46 (56.8)	7 (70)
III		35 (43.2)	3 (30)
IDH mutation at diagnosis			
Yes		43 (53.1)	6 (60)
No		25 (30.9)	3 (30)
Unknown		13 (16)	1 (10)
1p19q codeletion (or ATRX protein expression) at diagnosis			
Yes		15 (18.5)	4 (40)
No		47 (58)	3 (30)
Unknown		19 (23.5)	3 (30)
Surgery at diagnosis			
Biopsy alone		24 (29.6)	2 (20)
Partial resection		17 (21)	1 (10)
Subtotal resection		22 (27.2)	2 (20)
Total resection		15 (18.5)	4 (40)
Unknown		3 (3.7)	1 (10)
First radiation therapy			
Radiation therapy alone		36 (44.4)	5 (50)
Concomitant chemotherapy		41 (50.6)	4 (40)
Other ^(c)^		4 (5)	1 (10)
First chemotherapy			
Temozolomide		47 (58)	3 (30)
PCV		16 (19.8)	4 (40)
TEMOBIC		12 (14.8)	1 (10)
Other		5 (6.2)	2 (20)
Unknown		1 (1.2)	0 (0)
Second chemotherapy			
Temozolomide		31 (38.2)	3 (30)
PCV		16 (19.8)	0 (0)
Other		5 (6.2)	2 (20)
No second chemotherapy		29 (35.8)	5 (50)
**≥ 3 Chemotherapy lines**		23 (28.4)	3 (30)

(**a**) Median survival was 24 months (range: 14.1–103). (**b**) Missing information (tumor samples not available for histological reviewing). (**c**) Gamma knife stereotaxic radiosurgery, or missing details.

ATRX, alpha-thalassemia/mental retardation X-linked; BEV, bevacizumab; IDHm, isocitrate dehydrogenase mutated; IDHwt, IDH wild type; PCV, procarbazine, CCNU and vincristine; TEMOBIC, temozolomide and carmustine.

Before bevacizumab initiation, all patients had at least one surgical intervention and 26 (32.1%) underwent additional surgical interventions. Tumor stage was upgraded in 16 of these 26 patients (61.2%) compared with the initial diagnosis. All patients received chemotherapy (temozolomide for 58%, PCV for 19.8% and TEMOBIC [temozolomide-carmustine] for 14.8%) (40). Fifty-two (64.2%) patients received at least one other chemotherapy line, and 23 (28.4%) patients received three or more chemotherapy lines before bevacizumab. Forty-nine (60.5%) patients received chemotherapy before any radiation therapy. The radiotherapy received consisted of radiotherapy alone (44.4%) or in combination with chemotherapy (50.6%; temozolomide for 45.7%, bevacizumab for 2.5%, and temozolomide + bevacizumab for 1.2%). Nineteen (23.5%) patients received additional radiotherapy (median interval, 39.2 months after first radiotherapy).

### 3.2 Bevacizumab treatment

Bevacizumab was administered a dose of 15 mg/kg every 3 weeks in 67.9% of patients and a dose of 10 mg/kg every 2 weeks in 32.1% of patients. The median number of bevacizumab injections was 7 [range: 1–79]. Bevacizumab was associated with chemotherapy in 37 (45,7%) patients: temozolomide in 12 (32.4%) patients, CCNU or carmustine in 12 (32.4%) patients, irinotecan in 12 (32.4%) patients, and carboplatin in 1 patient (1.2%).

### 3.3 Bevacizumab toxicity

Severe adverse events (CTCAE grade ≥ 3) occurred in 10 (12.3%) patients: intra-tumoral hemorrhage (*n* = 4), nephrotic syndrome (*n* = 3), digestive hemorrhage (*n* = 1), digestive perforation (*n* = 1) and venous thrombosis (*n* = 1). Bevacizumab was discontinued in seven (8.6%) patients due to toxicity. No bevacizumab-related deaths were reported.

### 3.4 Tumor response and survival since bevacizumab initiation

According to the 2017 RANO criteria, the best response (whatever the initial tumor grade) was partial response in 22 (27.2%) patients, stable disease in 18 (22.2%) patients, and progressive disease in 41 (50.6%) patients. After disease progression during bevacizumab, 12 (14.8%) patients received another treatment (chemotherapy, radiotherapy, or surgical intervention) and 69 (85.2%) patients received best supportive care only.

In the univariate analysis, initial tumor proliferation index (Ki67) ≥ 7% (*OR* = 0.41, 95% CI 0.15-1.21, *p* = 0.079) tended to be inversely associated with partial response/stable disease (**Table 2**). None of the baseline patient characteristics (listed in **Table 1**) were associated with tumor response.

**Table 2:** Univariate and multivariate analyses to identify factors associated with tumor response, progression-free and overall survival since starting bevacizumab, and overall survival since diagnosis.

Factor	Best response (RANO criteria)^(a)(b)^		PFS since BEV initiation^(b)(c)^		OS since BEV initiation^(b)(c)^		OS since diagnosis^(c)^			
							Univariate		Multivariate	
	Odds ratio (95% CI)	*p*-value	Hazard ratio (95% CI)	*p*-value	Hazard ratio (95% CI)	*p*-value	Hazard ratio (95% CI)	*p*-value	Hazard ratio (95% CI)	*p*-value
**Age ≥ 38 years**	0.58 (0.24–1.39)	0.220	1.06 (0.67–1.66)	0.814	1.04 (0.66–1.64)	0.858	2.27 (1.4II–III.65)	**< 0.001**	1.95 (1.14–3.36)	**0.016**
**ICHT at diagnosis**	1.96 (0.49–7.75)	0.551	0.36 (0.17–0.81)	**0.021**	0.54 (0.26–1.13)	0.216	0.90 (0.44–1.83)	0.647	NS	NS
**Histological grade III**	0.47 (0.17–1.33)	0.153	1.06 (0.63–1.80	0.816	1.04 (0.61–1.76)	0.895	3.02 (1.69–5.39)	**< 0.001**	NS	NS
**Ki67 ≥ 7%^(d)^**	0.41 (0.15–1.12)	0.079	1.33 (0.80–2.22)	0.265	1.49 (0.88–2.50)	0.131	2.40 (1.41–4.09)	**< 0.001**	1.84 (1.04–3.22)	**0.031**
**IDHm**	1.87 (0.63–5.51)	0.225	0.80 (0.46–1.39)	0.427	0.80 (0.45–1.40)	0.435	0.29 (0.15–0.53)	**< 0.001**	NS	NS
**1p19q codeletion**	0.82 (0.26–2.54)	0.728	0.66 (0.36–1.21)	0.169	0.93 (0.52–1.67)	0.818	0.52 (0.28–0.97)	**0.035**	NS	NS
**Chemoradiotherapy^(e)^**	0.60 (0.25–1.46)	0.263	1.53 (0.96–2.44)	**0.043**	1.52 (0.95–2.43)	0.137	2.09 (1.31–3.35)	**0.008**	NS	NS
**≥3 chemotherapy lines**	0.58 (0.22–1.55)	0.273	1.15 (0.70–1.91)	0.580	1.17 (0.71–1.95)	0.539	0.50 (0.30–0.83)	**0.006**	NS	NS

Other patients baseline characteristics (**Table 1**) were tested, but no significant association was found with best response and survival.

^(a)^ Best response was defined as complete response, partial response, or stable disease (logistic regression). ^(b)^ Univariate analysis. ^(c)^ Cox proportional hazard model. ^(d)^ Ki67 threshold was defined as the median score in our cohort. ^(e)^ Stupp protocol and other protocols in which radiation therapy and chemotherapy are combined.

BEV, bevacizumab; ICHT, intracranial hypertension; IDHm, isocitrate dehydrogenase mutation; NS, not significant; OS, overall survival; PFS, progression-free survival; RANO, Response Assessment in Neuro-Oncology.

Bold values highlighted statistically significant p-values.

**Figure 1:**
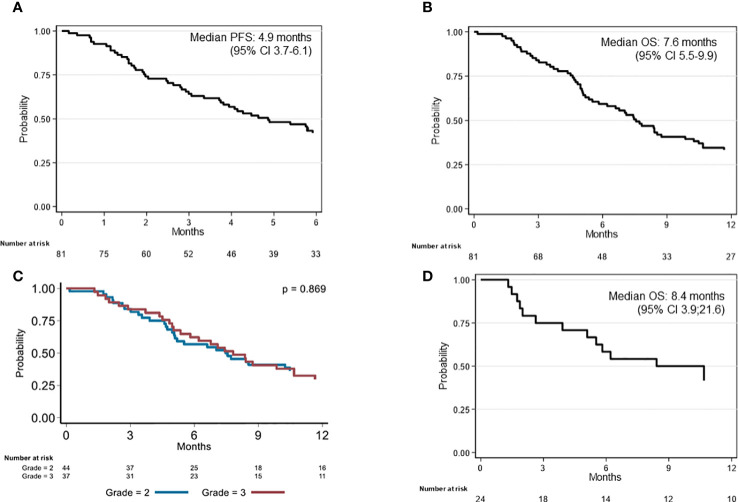
Kaplan–Meier analysis of survival in patients with recurrent grade II or III glioma on bevacizumab. **(A)** Progression-free survival (PFS) and **(B)** overall survival (OS) since bevacizumab initiation (whole sample; *n* = 81); **(C)** OS since bevacizumab initiation according to the WHO histological grade (whole samples); **(D)** OS since bevacizumab initiation of the 24 patients who discontinued treatment without radiological progression.

From the start of bevacizumab treatment, the median follow-up was 7.6 months and the median PFS was 4.9 months (95% CI 3.7–6.1) (**Figure 1A**). The 6-month PFS rate was 41.9% (95% CI 31.1–52.4). Intracranial hypertension at diagnosis (HR = 0.36, 95% CI 0.17–0.81, *p* = 0.021) and previous radiotherapy plus chemotherapy (HR = 1.53, 95% CI 0.96–2.44, *p* = 0.043) were significantly associated with PFS in the univariate analysis (**Table 2**), but not in the multivariate analysis. The median OS from bevacizumab initiation was 7.6 months (95% CI 5.5–9.9) (**Figure 1B**), and the 1-year OS rate was 33.3% (95% CI 23.4–43.6). None of the baseline characteristics were associated with OS in the univariate analysis (**Table 2**; **Figure 1C**).

The median follow-up since the time of diagnosis was 6.0 years. The median OS was 6.2 years (95% CI 4.6–7.8). OS was better in patients with WHO grade II gliomas at diagnosis than those with grade III (HR = 3.02, 95% CI 1.69–5.39, *p* < 0.001) (**Table 2**). In the multivariate analysis, Ki67 ≥ 7% (HR = 1.84, 95% CI 1.04–3.22, *p* = 0.031) and age at diagnosis ≥ 38 years (HR = 1.95, 95% CI 1.14–3.36, *p* = 0.016) were the only parameters associated with OS since disease diagnosis.

### 3.5 Subgroup analysis

**Figure 2:**
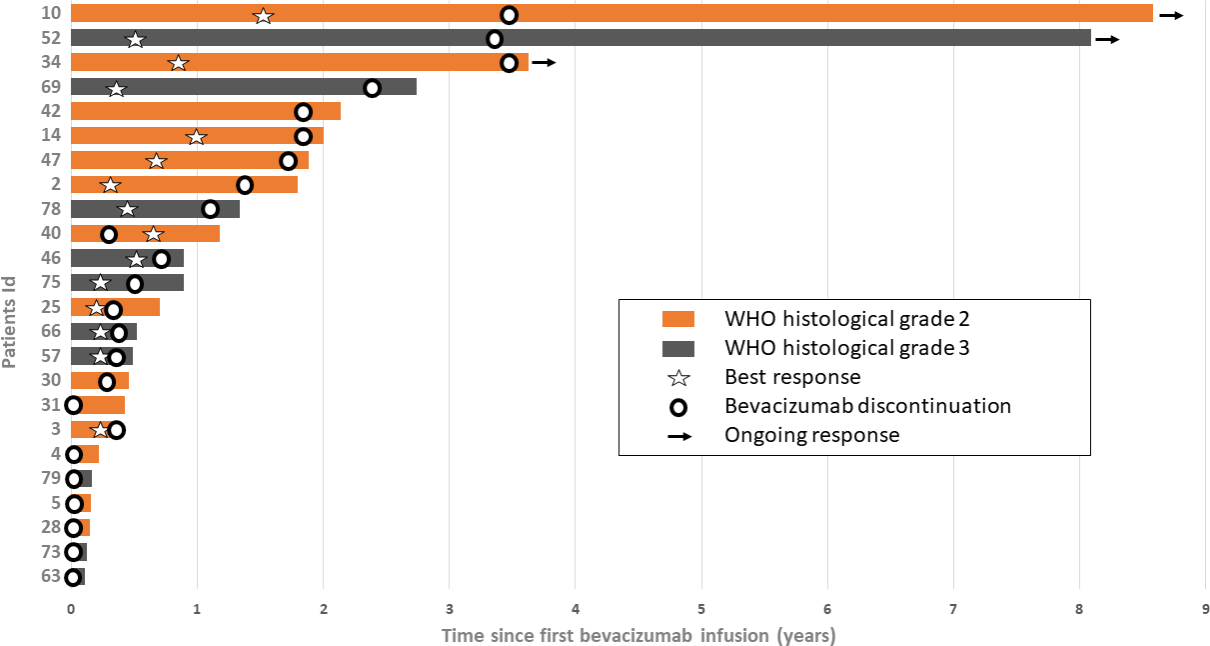
Survival since bevacizumab initiation in patients who discontinued bevacizumab without radiological progression (*n* = 24). Each bar corresponds to one patient. Bar length indicates the patient’s overall survival from bevacizumab initiation to death or last follow-up. Best response: stable disease or partial response.

Twenty-four (29.6%) patients stopped bevacizumab without radiological progression (**Figure 2**). Reasons for discontinuing bevacizumab were medical decision (*n* = 12), bevacizumab severe toxicity (*n* = 7), patient decision (*n* = 2), and unknown (*n* = 3). In the univariate analysis of predictive factors for discontinuing bevacizumab without radiological progression (**Table 3**), oligodendroglioma (1p19q codeletion) and age ≥ 38 years at diagnosis were associated with OR of 0.24 (95% CI 0.07-0.84, *p* = 0.023) and 0.36 (95% CI 0.13–0.99, *p* = 0.042), respectively. In this subgroup, the median OS since bevacizumab initiation was 8.4 months (95% CI 3.9–21.6) (**Figure 1D**) and the 12-month OS rate was 41.7% (95% CI 22.2–60.1).

**Table 3:** Univariate analysis of factors predictive of bevacizumab discontinuation without radiological progression.

Factor		No radiological progression (*n* = 24)	Radiological progression (*n* = 57)	Odds ratio	95% CI	*p*-value
Age at diagnosis						
< 38 years		8 (33.33)	33 (57.89)	1		**0.042**
≥ 38 years		16 (66.67)	24 (42.11)	0.36	0.13–0.99	
Symptoms at diagnosis						
Epilepsy		15 (65.22)	38 (69.09)	1		0.742
ICTH		4 (17.39)	6 (10.91)	0.59	0.15–2.40	
Other		4 (17.39)	11 (20.00)	1.09	0.30–3.95	
Histological WHO 2016 grade at diagnosis						
Grade II		9 (50.00)	20 (48.78)	1		0.931
Grade III		9 (50.00)	21 (51.22)	1.05	0.35–3.18	
Ki67 score ^(a)^						
< 7		9 (45.00)	18 (38.30)	1		0.610
≥ 7		11 (55.00)	29 (61.70)	1.32	0.46–3.80	
IDH mutation						
No		8 (47.06)	15 (38.46)	1		0.549
Yes		9 (52.94)	24 (61.54)	1.42	0.45-4.49	
1p19q codeletion						
No		6 (40.00)	28 (73.68)	1		**0.023**
Yes		9 (60.00)	10 (26.32)	0.24	0.07-0.84	
First surgery						
Biopsy alone		7 (31.82)	17 (30.36)	1		0.849
Subtotal/incomplete resection		10 (45.45)	29 (51.79)	1.19	0.38-3.72	
Total resection		5 (22.73)	10 (17.86)	0.82	0.21-3.30	
First radiation therapy						
Radiotherapy alone ^(b)^		15 (62.50)	24 (42.11)	1		0.106
Radio-chemotherapy		9 (37.50)	32 (56.14)	2.22	0.83-5.93	
Unknown		0 (0.00)	1 (1.75)	.	.	
Number of chemotherapy lines						
1/2		16 (66.67)	41 (73.21)	1		0.556
3/4		8 (33.33)	15 (26.79)	0.73	0.26-2.06	

Other patients’ baseline characteristics (**Table 1**) were tested, but no significant association was found.

(a) Ki67 threshold was defined as the median score in our cohort.

(b) Radiotherapy alone or radiosurgery.

BEV, bevacizumab; ICHT, intracranial hypertension; IDH, isocitrate dehydrogenase.

Bold values highlighted statistically significant p-values.

Among these 24 patients, 10 were still alive at 12 months (bevacizumab discontinuation due to severe toxicity in four, clinical degradation in three, and long treatment duration in three patients). Their survival ranged from 14.1 to 103 months (**Figure 2**). The median number of bevacizumab injections was 26.5 [range: 21–69]. Their baseline characteristics are summarized in **Table 1**. After bevacizumab discontinuation, eight patients did not receive any other chemotherapy, one patient received temozolomide again (2 months), and one patient received fotemustine (one injection). Two patients were alive 8 years after bevacizumab initiation. One was a 29-year-old man with a grade II astrocytoma IDHm (diagnosed in 2010). Before starting bevacizumab, he had received PCV (five cycles), radiotherapy alone (60 Gy), temozolomide (3 months), and had undergone a second total resection surgery (upgrading to grade III astrocytoma, with microvascular proliferation, and T1 gadolinium enhancement on MRI). Bevacizumab was started after surgery and was associated with irinotecan for seven injections, and then alone for 3 years. Bevacizumab was stopped for grade 3 high blood pressure associated with grade 2 proteinuria. This patient was still alive 8.6 years after bevacizumab initiation and 3.6 years after the last bevacizumab infusion. The other patient was a 48-year-old woman with a grade III astrocytoma IDHm (diagnosed in 2008). Total tumor resection was followed by radiotherapy (60 Gy) and adjuvant chemotherapy (PCV). At the first recurrence, the patient undergone radiosurgery. At the second recurrence, bevacizumab was started and continued for 30 months when it was discontinued in the absence of tumor progression. She was still alive at 8.1 years after bevacizumab initiation and 3.4 years after the last bevacizumab infusion. To date of last follow-up, both patients were still alive and have not received any subsequent treatment.

## 4 Discussion

This retrospective study evaluated bevacizumab efficacy in heavily pretreated patients (64.2% had received two or more systemic chemotherapy lines, 32.1% had undergone a second surgical intervention, and 23.5% had undergone two or more radiotherapy treatments). All patients had baseline T1 gadolinium enhancement on MRI, highlighting tumor neovascularization or necrosis and the possible tumor upgrading (41). Indeed, progression to higher histological grade was observed in 61.5% of patients after surgical reintervention.

The median PFS and OS since bevacizumab initiation were 4.9 (95% CI 3.7–6.1) and 7.6 months (95% CI 5.5–9.9), respectively, in line with previous studies on oligodendroglioma, high-grade gliomas and also on advanced glioblastomas (14, 23, 24). This suggests that the duration of the response to bevacizumab is about the same magnitude whatever the WHO tumor grade and whatever its position in the patients’ management strategy. Bevacizumab toxicity profile was as previously reported (13–15).

In our study, IDHm, 1p19q codeletion (or ATRX protein expression), percentage of Ki67-positive cells and histological grade were associated with survival of patients with recurrent grades II–III glioma from diagnosis, but not from bevacizumab initiation. Oligodendroglioma IDHm/1p19q codeletion, grade II gliomas, and low Ki67 are known good prognostic factors (2, 36, 42). However, in our sample, they were not predictive of bevacizumab efficacy. This may be related to disease progression toward glioma transformation, as indicated by T1 contrast enhancement and upgrading in reoperated patients. These patients’ tumors may have the same evolution as grade IV gliomas; however, 1p19q codeletion had no impact on OS after bevacizumab. Moreover, our study failed to demonstrate a relationship between the efficacy of bevacizumab and IDHm. This could be related to missing data and lack of statistical power. Only intracranial hypertension at diagnosis was associated with PFS since bevacizumab initiation. This could be explained by bevacizumab antiangiogenic activity related to neovascularization that induces cerebral edema and intracranial hypertension. Our multivariate analysis did not find any association between patient characteristics and OS. This suggests that patient response to bevacizumab and survival since starting bevacizumab are not correlated with the initial histological tumor grade or mutational status.

In addition, a subgroup of patients discontinued bevacizumab without radiological progression. Oligodendroglioma and age ≥ 38 years at diagnosis were associated with this subgroup and might be a biomarker for discontinuing bevacizumab without radiological progression. It is known that survival is longer in patients with oligodendrogliomas than with astrocytomas (2). Conversely, age ≥ 38 years at diagnosis was an unexpected association as older age is usually negatively associated with survival (2, 13). We may hypothesize that these patients, who had been on heavy treatment before starting bevacizumab, were more frail and were more prone to discontinuing bevacizumab due to clinical degradation, bevacizumab toxicity, or presence of comorbidities that were incompatible with continuing bevacizumab. Indeed, the main reasons for discontinuing bevacizumab in this subgroup were patient/physician decision and severe toxicity. Among these 24 patients, 10 were alive at 12 months. Most of these long-term responders did not receive any other treatment after bevacizumab. Moreover, two patients were still alive 8 years after initiating bevacizumab as a rescue treatment. A previous study also reported long-term survivors among patients on bevacizumab for recurrent grades II–III gliomas (34). However, to the best of our knowledge, this is the first report of maintained bevacizumab response after treatment discontinuation without radiological progression in patients with recurrent grades II–III glioma. This long-term effect might be explained by bevacizumab’s capacity to activate the anti-tumor immune response by promoting the infiltration of anti-tumor lymphocytes and decreasing the pro-tumor lymphocyte infiltrate (43–46). In agreement, in patients with glioblastoma on bevacizumab, an increase in inflammatory markers in peripheral blood has been associated with better survival (47). Different mechanisms may be involved, such as promotion of diapedesis, maturation of dendritic cells, a decrease in myeloid suppressor cell infiltration, or the regulation of glutamate transport (45, 46). Alternatively, bevacizumab’s inhibitory effects on the VEGFR pathway (angiogenesis) and on glioma stem cells might be implicated. Pathways leading to bevacizumab resistance (e.g., *cMET* upregulation, induction of other neo-angiogenic pathways) and their regulation also should be investigated (48, 49).

Our study has certain limitations. First, the retrospective study design limits the extrapolation of our results. Second, some data were missing (particularly IHC data), and tumor methylation profiles were not investigated. Moreover, IDH gene sequencing was only available for a few patients’ and was not reported. Third, the 2016 WHO classification has been replaced by a new WHO classification (50). However, the distinction between grades II and III glioma has not been fundamentally changed. The only exceptions are also the presence of *CDKN2A/B* homozygous deletion for IDHm astrocytoma, and of *TERT* promoter mutation, *EGFR* gene amplification, and chromosome 7 gain/chromosome 10 loss for IDH wild-type diffuse astrocytomas, which classify them as grade IV, which were missing in our study. However, the IHC analysis at inclusion gave similar IDHm and 1p19q codeletion rates as previously reported (51). Fourth, our patient cohort, with its distinct molecular pattern and bevacizumab administration, was not homogeneous. Half patients had undergone chemotherapy combined with bevacizumab and bevacizumab was administered at 10 or 15 mg/kg every 2 or 3 weeks. However, that both administration schemas are not different in terms of treatment efficacy (52). Finally, the RANO high-grade glioma criteria were used, based on the assumption that the study sample included mainly patients with transformed diffuse gliomas. In the vast majority of the cases, the increasing of enhancement was followed by spread of the infiltration of the tumor. In the other cases, FLAIR hyper-signal was considered as stable. However, radiological progression was assessed by MRI every 3 months. Considering PFS on radiological evaluation was a limitation. Indeed, among patients who discontinued bevacizumab without radiological progression, application of the RANO criteria would probably have enabled them to be classified as progressive solely on the criterion of clinical degradation (39).

## 5 Conclusion

Bevacizumab alone or in combination can be proposed as a rescue treatment for heavily treated patients with recurrent grades II–III glioma and T1 contrast enhancement on brain MRI. PFS and OS were the same as reported for glioblastomas. In our study, the response to bevacizumab was not associated with the histological grade or tumor biology at diagnosis. Few patients who responded to bevacizumab experienced prolonged survival after discontinuing bevacizumab, without any subsequent treatment. We now need to identify the clinical, biological, and/or radiological factors that predict the long-term response to bevacizumab in these patients.

## Data availability statement

The raw data supporting the conclusions of this article will be made available by the authors, without undue reservation.

## Ethics statement

Ethical review and approval was not required for the study on human participants in accordance with the local legislation and institutional requirements. Written informed consent for participation was not required for this study in accordance with the national legislation and the institutional requirements.

## Author contributions

All authors contributed to the study conception and design. Material preparation, data collection and analysis were performed by SA, MJ, AD, and MF. The first draft of the manuscript was written by SA. and all authors commented on previous versions of the manuscript. All authors contributed to the article and approved the submitted version.
